# RNAi reveals proteins for metabolism and protein processing associated with Langat virus infection in *Ixodes scapularis* (black-legged tick) ISE6 cells

**DOI:** 10.1186/s13071-016-1944-0

**Published:** 2017-01-13

**Authors:** Jeffrey M. Grabowski, Monika Gulia-Nuss, Richard J. Kuhn, Catherine A. Hill

**Affiliations:** 1 Department of Entomology, College of Agriculture, Purdue University, 901 W State Street, West Lafayette, IN 47907 USA; 2 Markey Center for Structural Biology, Department of Biological Sciences, College of Science, Purdue University, 915 W State Street, West Lafayette, IN 47907 USA; 3 Purdue Institute for Inflammation, Immunology and Infectious Disease, Purdue University, West Lafayette, IN 47907 USA; 4 Current Address: NIH/NIAID, Rocky Mountain Laboratories, Laboratory of Virology, Biology of Vector-Borne Viruses Section, 903 S 4th St, Hamilton, MT 59840 USA; 5 Current Address: Department of Biochemistry and Molecular Biology, College of Agriculture, Biotechnology, and Natural Resources, University of Nevada-Reno, 1664 N Virginia Street, Reno, NV 89503 USA

**Keywords:** *Ixodes scapularis*, Tick-borne disease, Flavivirus, Langat, ISE6, RNAi, Functional studies, Metabolism, Protein-processing

## Abstract

**Background:**

Tick-borne flaviviruses (TBFs) cause thousands of human cases of encephalitis worldwide each year, with some TBF infections progressing to hemorrhagic fever. TBFs are of medical and veterinary importance and strategies to reduce flavivirus transmission by the tick vector may have significant application. Analyses of the proteome of ISE6 cells derived from the black legged tick, *Ixodes scapularis* infected with the TBF, Langat virus (LGTV), have provided insights into proteins and cellular processes involved with LGTV infection.

**Methods:**

RNA interference (RNAi)-induced knockdown of transcripts was used to investigate the role of ten tick proteins in the LGTV infection cycle in ISE6 cells. LGTV-infected cells were separately transfected with dsRNA corresponding to each gene of interest and the effect on LGTV genome replication and release of infectious virus was assessed by RT-qPCR and plaque assays, respectively.

**Results:**

RNAi-induced knockdown of transcripts for two enzymes that likely function in amino acid, carbohydrate, lipid, terpenoid/polykeytide and vitamin metabolism, and a transcript for one protein of unknown function were associated with decreased replication of the LGTV genome and release of infectious virus from cells. The knockdown of transcripts for five enzymes predicted to function in metabolism, a protein likely associated with folding, sorting and degradation, and a protein of unknown function was associated with a decrease only in the amount of infectious LGTV released from cells.

**Conclusions:**

These data suggest tick proteins potentially associated with metabolism and protein processing may be involved in LGTV infection of ISE6 cells. Our study provides information to begin to elucidate the function of these proteins and identify targets for the development of new interventions aimed at controlling the transmission of TBFs.

**Electronic supplementary material:**

The online version of this article (doi:10.1186/s13071-016-1944-0) contains supplementary material, which is available to authorized users.

## Background

Tick-borne flaviviruses (TBFs) impact human and animal health worldwide. These positive, single-stranded RNA viruses are transmitted by an infected tick (subphlym Chelicerata, subclass Acari, family Ixodidae) during blood-feeding. Tick-borne encephalitis virus (TBEV), Powassan virus (POWV), Kyasanur Forest Disease virus (KFDV) and Omsk hemorrhagic fever virus (OHFV) are members of the TBF complex and can cause encephalitis (TBEV and POWV) and hemorrhagic fever (KFDV and OHFV). Many TBFs are considered a biosecurity threat and are classified as biosafety containment level (BSL) three or four. Research is ongoing to develop vaccines and therapeutics to prevent or treat TBF infections. The less virulent Langat virus (LGTV), classified BSL-2, has been widely used as a model for more virulent TBFs. Many studies have employed LGTV and cell lines derived from the black-legged tick, *Ixodes scapularis* to investigate viral pathogenesis in the tick host cell, although *I. scapularis* is not a natural vector of LGTV.

Studies to understand the pathogenesis of tick-virus interactions will benefit from the recently published assembly of the *I. scapularis* genome. As the first such resource for a tick vector of disease [1–3], the assembly is expected to help advance investigations of tick-virus interactions at the molecular level. Proteomic studies have identified hundreds of *I. scapularis* proteins [4–7] and helped to define the proteome of this vector. Using mass spectrometry, Grabowski et al. identified 486 proteins in the *I. scapularis* ISE6 cell line, 266 of which were differentially regulated in cells infected with LGTV [4]. Proteins likely associated with metabolic processes exhibited increased or decreased expression following LGTV infection. These and other studies [7–9] provide a logical starting point for detailed molecular research to determine the role of tick proteins during the TBF life-cycle in the vector.

RNA interference (RNAi) is a tool widely used for functional studies of arthropod proteins, including proteins produced by the *I. scapularis* IDE8 cell line during infection with flavivirus [7–9]. At least one of these studies suggests induction of the RNAi-based antiviral pathway identified in other organisms and a role for Argonaute and Dicer in suppression of LGTV genome replication [9], although a role for the RNAi-pathway protein, Tudor-SN in LGTV replication or release of infectious virus is questioned [8]. Other IDE8 gene products implicated in the antiviral response of the tick cell against LGTV include Factor H, trypsin, HSP90 and HSP70 [7], with the latter two proteins predicted to function in protein folding and/or processing. Previous studies shed light on lipids and metabolic processes potentially manipulated by dengue virus (DENV) to facilitate infection and replication in human and mosquito systems [10–12]. Equivalent studies are required to better understand metabolic processes affected during tick-flavivirus interaction.

Efforts are underway to develop new transmission-blocking technologies that target proteins produced by the host cell (i.e. host factors) that are essential to virus infection and replication [13–16]. Here, we investigated the hypothesis that proteins which exhibited increased expression in LGTV-infected *I. sapularis* ISE6 cells and are predicted to function in (i) the metabolism of amino acids, vitamins/cofactors, carbohydrates, nucleotides and lipids, (ii) DNA replication/repair or (iii) protein folding/sorting/degradation [4] are involved in flaviviral infection. The functional roles performed by these proteins during LGTV infection was pursued using loss-of-function, RNAi knockdown assays. Ten genes of interest were selected for analyses: fumarylacetoacetase (FAH; ISCW020196), endoplasmic reticulum protein 29 (ERP29; ISCW018425), aldehyde dehydrogenase (ALDH; ISCW015982), carbon-nitrogen hydrolase/pantetheine hydrolase/vanin-like (VNN; ISCW004822), malate dehydrogenase (MDH2; ISCW003528), poly [ADP-ribose] polymerase (PARP; ISCW019519), cytidine/uridine monophosphate kinase (CMPK; ISCW012446), acetyl-CoA acetyltransferase (ACAT1; ISCW016117) and two hypothetical proteins (Hypo195; ISCW011195 and Hypo576; ISCW020576). The process used to select these ten genes is summarized in Additional file 1: Figure S1. Transcripts corresponding to the above genes were confirmed in ISE6 cells and adult *I. scapularis*. Subsequently, ISE6 cells were separately transfected with dsRNA corresponding to each gene of interest, the knockdown of transcripts was confirmed by reverse transcriptase quantitative PCR (RT-qPCR) and the effect on LGTV genome replication and release of infectious virus was assessed by RT-qPCR and plaque assay, respectively. Knockdown of transcripts for VNN, ACAT1, and Hypo576 was associated with decreased LGTV genome replication and LGTV release, while knockdown of transcripts for FAH, ERP29, ALDH, MDH2, CMPK, and Hypo195 was associated with decreased LGTV release only. These proteins are candidates for further functional analyses and studies aimed at development of new technologies to prevent transmission of TBF infection.

## Methods

### Cell and LGTV culture

The ISE6 cell line derived from *I. scapularis* embryonic cells (obtained from T. Kurtti, University of Minnesota, Minneapolis, MN) was cultured at 34 °C in L15B-300 medium in the absence of CO_2_ [17, 18]. Baby hamster kidney 15 cells (BHK15) and Green African monkey kidney cells (Vero) originally obtained from American Tissue Culture Collection (ATCC), were cultured at 37 °C in Minimum Essential Medium (MEM) supplemented with L-glutamine, non-essential amino acids, and 10% heat-inactivated fetal calf serum (FCS) with 5% CO_2_. LGTV TP21 wildtype strain (passage 2) was obtained from A. Pletnev (NIH-NAID, Bethesda, MD) and amplified using a multiplicity of infection (MOI) of 0.01 [19, 20] in Vero cells (cells were grown as described above, with 2.5% heat-inactivated FCS) to produce a working stock (passage 4). The titer of the LGTV p4 stock was determined *via* serial immunofluorescence assays (IFAs) in Vero cells using expression of the LGTV nonstructural protein 3 (NS3) as described by Junjhon et al. [21] and Grabowski et al. [4]. LGTV infection of ISE6 cells was performed using an MOI of 10 to achieve maximal infection of the cell population [4] and capture the synchronized release of the first population of infectious virus. Manual cell counts (cells/ml) were conducted using a hemocytometer [22] to quantify cell numbers before (a) seeding and (b) infection with LGTV.

### Preparation of RNA from adult *I. scapularis* and ISE6 cells and cDNA synthesis

A single *I. scapularis* female collected by flagging from Tippecanoe State Park, Winamac, IN (October 29, 2013) was flash frozen in liquid N_2_ and ground in TRIzol reagent (Invitrogen, Carlsbad, USA) using mortar and pestle, and RNA was extracted as per manufacturer instructions. RNA was isolated from ISE6 cells (passage 96–100) grown in 96 well plates using the RNeasy mini kit (Qiagen, Hilden, Germany) and processed according to kit instructions. cDNA was synthesized from RNA samples using the iScript cDNA synthesis kit (BioRad, Hercules, USA). Thermocycler conditions used for cDNA synthesis were as follows: 25 °C for 5 min, 42 °C for 50 min, and 85 °C for 5 min.

### Confirmation of transcripts for genes of interest in adult *I. scapularis* and ISE6 cells

Primers were designed using Primer3 software [23, 24] and NCBI Primer-BLAST [25] (http://www.ncbi.nlm.nih.gov/tools/primer-blast/). GenBank accession numbers for the genes of interest are as follows: fumarylacetoacetase (FAH; XP_002407463), endoplasmic reticulum protein 29 (ERP29; XP_002435676), aldehyde dehydrogenase (ALDH; XP_002399265), carbon-nitrogen hydrolase/pantetheine hydrolase/vanin-like (VNN; XP_002402506), malate dehydrogenase (MDH2; XP_002402153), poly [ADP-ribose] polymerase (PARP; XP_002409668), cytidine/uridine monohydrate kinase (CMPK; XP_002413690), acetyl-CoA acetyltransferase (ACAT1; XP_002402965), hypothetical protein (Hypo195; XP_002411582), and hypothetical protein (Hypo576; XP_002408828). Primers were designed to amplify products ranging from 300 to 607 bp and spanning at least one intron (Additional file 1: Table S1).

PCR was conducted using Phusion high-fidelity PCR master mix with HF buffer (NE Biolabs, Ipswich, USA), cDNA template prepared from female *I. scapularis* or ISE6 cell RNA, and the following thermocycler conditions: 94 °C for 5 min; 32 cycles of 94 °C for 30 s, 58 °C for 30 s and 72 °C for 2 min, and 72 °C for 7 min. Following gel electrophoresis on 1.5% TBE agarose gel, amplicons of expected size were excised and purified using the Qiagen gel extraction kit (Qiagen) and the sequence was confirmed *via* direct sequencing at the Purdue Genomics Core Facility (PGCF), Purdue University.

### Synthesis of dsRNA corresponding to *I. scapularis* genes of interest

T7-tagged cDNA template was generated using cDNA prepared from ISE6 cell RNA and T7-tagged primers (Additional file 1: Table S1). Two-step PCR was performed using the following thermocycler conditions: 94 °C for 5 min; 5 cycles at 94 °C for 30 s, 58 °C for 30 s, 72 °C for 2 min; 27 cycles at 94 °C for 30 s, 68 °C for 30 s, 72 °C for 2 min, and final extension at 72 °C for 7 min. dsRNA was synthesized from T7-tagged cDNA template using the MEGAscript RNAi synthesis kit according to manufacturer’s instructions and Barry et al. [26]. Conceptual cDNA sequences were searched by BLASTn against the *I. scapularis* IscaW1.4 transcript dataset at VectorBase (https://www.vectorbase.org/) to confirm the specificity of expected siRNA products for the target gene (i.e. to limit off-target effects).

### Transfection of ISE6 cells with dsRNA and transcript knockdown

ISE6 cells were seeded at ~ 1 × 10^5^ cells per well in 96-well flat-bottom cell culture plates pretreated with poly-L-lysine (Sigma Aldrich, St. Louis, USA). For dsRNA transfections, ISE6 cells were cultured for 48 h, following which half of the media was removed from each well and replaced with an equal volume of transfection mix (OptiMEM Reduced Serum Medium, Glutamax supplement [Invitrogen] and X-tremeGENE siRNA transfection reagent [Roche, Basel, Switzerland] prepared according to [26]) and 10 ng dsRNA per well. Cells were incubated for 60 h, following which media/transfection mix was removed and cells were incubated with resazurin salt (Sigma Aldrich) complete tick media (0.275 mM final concentration) for 12 h and cell viability was assessed *via* fluorescent readout on a Molecular Devices SpectraMax M5 plate reader coupled with SoftMax Pro v4.8 software (excitation at 560nm, emission at 590 nm) as described in Grabowski et al. [4]. In parallel, RNA was extracted from cells collected at 60 h post-transfection using RLT lysis buffer (from Qiagen RNeasy kit) to confirm knockdown of transcripts.

To assess the effect of dsRNA-mediated knockdown on replication of the LGTV genome, media/transfection mix was removed at 60 h post-transfection, cells were infected with LGTV (1 h rocking adsorption at room temperature), rinsed 3 times with 1× PBS, and incubated with fresh media for 12 h, following which RNA was extracted using RLT buffer for RT-qPCR analyses.

To assess the effect of dsRNA-mediated knockdown on the release of infectious LGTV, cells were either transfected with dsRNA (i) prior to (pre-treatment with dsRNA) or (ii) immediately following (post-treatment with dsRNA) LGTV infection. For pretreated cells, media/transfection mix was removed at 60 h post-transfection, cells were infected with LGTV (as above), rinsed 3 times with 1× PBS and incubated with fresh media for 16 h post-infection (hpi), following which media was collected for plaque assays. Post-treated cells were first infected with LGTV as above, rinsed three times with 1× PBS, incubated with media/transfection mix for 60 h and media was collected for plaque assays. Unpaired, two-tailed t-test statistical analyses were performed with GraphPad Prism (v4.03) software.

The amount of RNA extracted per 96-well following dsRNA transfections or LGTV infection ranged from 5.8 to 29.9 ng/μl. To confirm dsRNA-mediated knockdown of mRNA, the relative levels of ISE6 cell transcripts were determined using the Quantifast SYBR Green PCR kit (Qiagen) and RT-qPCR primers (Additional file 1: Table S2) relative to the *I. scapularis β-actin* gene. Primers targeting the negative strand of the replicative LGTV genome intermediate were used to quantify LGTV transcripts as described by Mitzel et al. [27] as a measure of LGTV genome replication relative to the *β-tubulin* gene. Reactions were performed on the Applied Biosystems 7300 PCR system (Life Technologies, Carlsbad, USA) in MicroAmp Optical 96-well reaction plates with labeled barcode (Life Technologies). The SDS RQ study software (v1.4.1) was used to collect raw C_t_ cycle values and the Comparative C_T_ Method (ΔΔCt Method) [28, 29] was used to determine relative transcript expression and an unpaired, two-tailed t-test was performed with GraphPad Prism (v4.03) software.

### Analysis of proteins using the DAVID functional clustering software

The annotation tool DAVID (http://david.abcc.ncifcrf.gov/ ) [30], was used to assign putative biological function to clusters of ISE6 proteins that exhibited increased expression following incubation with LGTV (LGTV), UV inactivated LGTV (UV-LGTV) or both (LGTV & UV-LGTV) identified in Grabowski et al. [4]. The GenBank accessions of orthologous proteins that mapped to *I. scapularis* KEGG pathways were used as input. Gene Ontology (GO) options were selected as output for DAVID clustering and an enrichment score of “≥1.3 is equal to a *P*-value ≤ 0.05” was used as cut-off. For each cluster, a modified Fisher’s exact test *P*-value ≤ 0.05 was used as an additional cut-off.

### Prediction of protein-protein interactions using STRING

STRING (v9.1; string91.embl.de; [31, 32]) was used to predict protein-protein interactions for the conceptual products of the 10 genes analyzed in this study. Binding partners were predicted for each gene product using VectorBase accession ID as input and a cut-off score of ≥ 0.70 (high confidence score).

## Results

### Selection and characterization of *I. scapularis* genes of interest for dsRNA-mediated knockdown of ISE6 transcripts

Ten genes of interest were selected based on (i) evidence of increased protein expression following LGTV infection, (ii) quality of the protein identification data from LC-MS/MS (proteins supported by ≥ 2 peptides), and (iii) orthology to vertebrate/invertebrate proteins (based on KEGG) [4] (Additional file 1: Figure S1). The genes were fumarylacetoacetase (FAH; VectorBase accession ID ISCW020196), secreted protein (ERP29; ISCW018425), aldehyde dehydrogenase (ALDH; ISCW015982), carbon-nitrogen hydrolase (VNN; ISCW004822), malate dehydrogenase (MDH2; ISCW003528), poly [ADP-ribose] polymerase (PARP; ISCW019519), cytidine/uridine monophosphate kinase (CMPK; ISCW012446), acetyl-CoA acetyltransferase (ACAT1; ISCW016117), hypothetical protein (Hypo195; ISCW011195), and hypothetical protein (Hypo576; ISCW020576) (Table 1).

**Table 1:** Summary of *Ixodes scapularis* genes selected for RNAi analyses

Tick Protein; KEGG^a^ Entry	*I. scapularis* VectorBase accession ID	GenBank accession number	Predicted function	% amino acid identity to *H. sapien*s ortholog	Location of gene on IscaW1 (scaffold: bp range)	Number of paralogs identified in IscaW1 assembly	Predicted number of protein binding partners^b^
Fumarylacetoacetase (FAH)	ISCW020196	XP_002407463	Amino acid metabolism	65.9	DS831757: 33,311–44,738	–	2
Endoplasmic reticulum protein 29 (ERP29)	ISCW018425	XP_002435676	Protein folding, sorting, & degradation	17	DS758338: 84,585–88,966	–	0
Aldehyde dehydrogenase (ALDH)	ISCW015982	XP_002399265	Amino acid metabolism	56.7	DS612682: 208,981–240,639	16	6
Carbon-nitrogen hydrolase/pantetheine hydrolase/vanin-like (VNN)	ISCW004822	XP_002402506	Metabolism of cofactors & vitamins	33.9	DS712062: 22,027–47,303	–	0
Malate dehydrogenase (MDH2)	ISCW003528	XP_002402153	Carbohydrate metabolism	68.9	DS711115: 47,872–64,175	–	21
Poly [ADP-ribose] polymerase (PARP)	ISCW019519	XP_002409668	DNA replication & repair	48.6	DS807313: 23,492–74,749	2	30
Cytidine/uridine monophosphate kinase (CMPK)	ISCW012446	XP_002413690	Nucleotide metabolism	40.2	DS915558: 7422–14,364	3	2
Acetyl-CoA C-acetyltransferase (ACAT1)	ISCW016117	XP_002402965	Carbohydrate, lipid, amino acid, terpenoid/polykeytide metabolism	60.2	DS624476: 15,968–41,821	2	24
Hypothetical protein (Hypo195)	ISCW011195	XP_002411582	Unknown	–	DS857119: 268,751–271,378	–	–
Hypothetical protein (Hypo576)	ISCW020576	XP_002408828	Unknown	–	DS835548: 71,893–118,756	–	–

^a^Kyoto Encyclopedia of Genes and Genomes; http://www.genome.jp/kegg/

^b^Number of potential protein binding partners as predicted *via* STRING

The DAVID cluster analyses of biological function provided insight into the response of ISE6 cells to infection with LGTV and UV-LGTV. ISE6 cell proteins reported in Grabowski et al. [4] that exhibited increased expression following exposure to LGTV or UV-inactivated LGTV (i.e. assigned to one or more of the following four datasets: LGTV, UV-LGTV, LGTV/UV-LGTV or LGTV & UV-LGTV) and mapped to KEGG pathways, were used to identify “clusters of biological processes” (Additional file 1: Table S3). Of the 10 ISE6 proteins selected for knockdown studies, MDH2 and PARP were identified in the cluster “translation, ribosomal function, and protein metabolic processing”, FAH, ALDH, and CMPK in both “nitrogen metabolic processing” and “nitrogen/amine/amino acid metabolic processing” clusters, and ACAT1 in the cluster “nitrogen metabolic processing function”. FAH, ALDH, MDH2, PARP, and CMPK were also identified in the cluster “ribonucleoprotein/ribosomal/translation/protein metabolic function.”

Sequence similarity analyses revealed that four of the 10 proteins (ERP29, VNN, PARP, and CMPK) had < 50% amino acid identity to the human ortholog (Table 1). Searches of the *I. scapularis* genome revealed no evidence of paralogs for the genes *FAH, ERP29, VNN, MDH2, Hypo195*, and *Hypo576*. Analyses suggest that *ALDH, PARP, CMPK*, and *ACAT1* are members of multi-gene families comprising 16, 2, 3, and 2 genes respectively (Table 1). STRING predicted multiple binding partners for PARP (30), ACAT1 (24), MDH2 (21), ALDH (6), FAH (2), and CMPK (2) in *I. scapularis,* suggesting these proteins may be associated with multiple protein-protein interactions and cellular processes. Binding partners were not predicted for ERP29 and VNN in *I. scapularis*.

Transcripts for the 10 genes of interest amplified from a single female *I. scapularis* or the ISE6 cell line had a minimum of 96% nucleotide identity to the corresponding IscaW1 gene model (Additional file 1: Table S4), suggesting significant conservation between the genome of the ISE6 cell line, field collected material, and the Wikel reference strain used to produce the IscaW1 genome assembly and annotation.

### Confirmation of dsRNA-mediated knockdown of transcripts for genes of interest in ISE6 cells

**Fig. 1:**
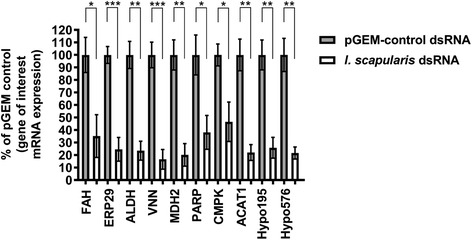
dsRNA-mediated knockdown of transcripts for *I. scapularis* genes of interest in ISE6 cells. Following transfection of ISE6 cells with 10 ng dsRNA for 60 h, total RNA was prepared from ~ 1 × 10^5^ cells and cDNA was amplified via a two-step RT-PCR reaction. mRNA levels were normalized to *I. scapularis β-actin* and expressed relative to the percentage of pGEM control cDNA. Results show relative expression of ten *I. scapularis* genes of interest following knockdown (*white* bars) relative to the pGEM dsRNA negative control (*gray* bars). Error bars represent standard error of the mean (SEM). Statistical analysis was performed using an unpaired t-test between the negative pGEM control and dsRNA treatments for each gene of interest. Results represent 2–3 technical replicates (each with 2–3 machine replicates) and 2 biological replicates. *P ≤ 0.05; **P ≤ 0.01; ***P ≤ 0.001. *Abbreviations*: FAH, fumarylacetoacetase (ISCW020196); ERP29, endoplasmic reticulum protein 29 (ISCW018425); ALDH, aldehyde dehydrogenase (ISCW015982); VNN, carbon-nitrogen hydrolase/vanin-like (ISCW004822); MDH2, malate dehydrogenase (ISCW003528); PARP, poly [ADP-ribose] polymerase (ISCW019519); CMPK, cytidine/uridine monophosphate kinase (ISCW012446); ACAT1, acetyl-CoA acetyltransferase (ISCW016117); Hypo195, hypothetical protein (ISCW011195); Hypo576, hypothetical protein (ISCW020576); pGEM, pGEM plasmid (negative control)

*In vitro* studies using pGEM dsRNA revealed that 10 or 25 ng dsRNA had no significant effect (*P* ≤ 0.05) on viability of ISE6 cells (Additional file 1: Figure S2, Table S5). Subsequent RNAi knockdown experiments were conducted using 10 ng dsRNA and *in vitro* studies revealed no significant effect (*P* ≤ 0.05) of dsRNA treatment on cell viability of RNAi-treated cells (Additional file 1: Figure S3). In parallel, knockdown of transcripts for all ten genes of interest was confirmed using RT-qPCR (Fig. 1).

### Effect of dsRNA-mediated knockdown of transcripts on LGTV infection

**Fig. 2:**
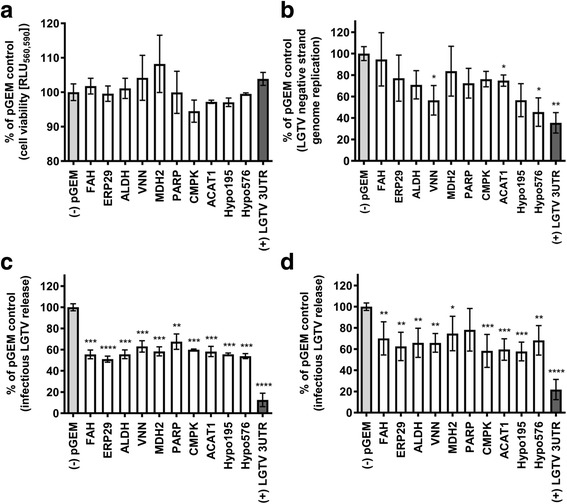
Effect of knockdown of *I. scapularis* transcripts on LGTV infection of ISE6 cells. **a** ISE6 cell viability following transfection with 10ng dsRNA for 60 h and 12 h post-infection (hpi) with LGTV, compared to the pGEM negative control. Results represent 2–5 technical replicates and 3 biological replicates. **b** Effect of *I. scapularis* transcript knockdown on LGTV genome replication in ISE6 cells following transfection with 10ng dsRNA for 60 h and 12 hpi with LGTV as compared to the negative control. Comparison of fold change in transcripts for the LGTV negative strand were normalized to the *I. scapularis β-tubulin* gene and expressed relative to percentage of pGEM control. Results represent 2 technical replicates (each with 3 machine replicates) and 2 biological replicates. **c** Effect of *I. scapularis* transcript knockdown on release of infectious LGTV from ISE6 cells transfected with dsRNA for 60 h prior to LGTV infections as assessed by plaque assay and normalized to the pGEM dsRNA negative control. Results represent 5 technical replicates and 2 biological replicates. **d** Effect of *I. scapularis* transcript knockdown on release of infectious LGTV from ISE6 cells infected with LGTV and transfected with dsRNA for 60 h. Results represent 2–5 technical replicates and 3 biological replicates. Error bars represent SEM for unpaired t-tests comparing cell viability, negative strand levels or pfu/ml of the negative pGEM control *versus* each gene of interest. *P ≤ 0.05; **P ≤ 0.01; ***P ≤ 0.001. *Abbreviations*: FAH, fumarylacetoacetase; ERP29, endoplasmic reticulum protein 29; ALDH, aldehyde dehydrogenase; VNN, carbon-nitrogen hydrolase/vanin-like; MDH2, malate dehydrogenase; PARP, poly [ADP-ribose] polymerase; CMPK, cytidine/urdine monophosphate kinase; ACAT1, acetyl-CoA acetyltransferase; Hypo195 and Hypo576, hypothetical proteins 195 and 576; pGEM, pGEM plasmid negative control (*light gray* bars); LGTV 3UTR, 3’ UTR of LGTV positive control (*dark gray bars*); RLU, relative light units

**Fig. 3:**
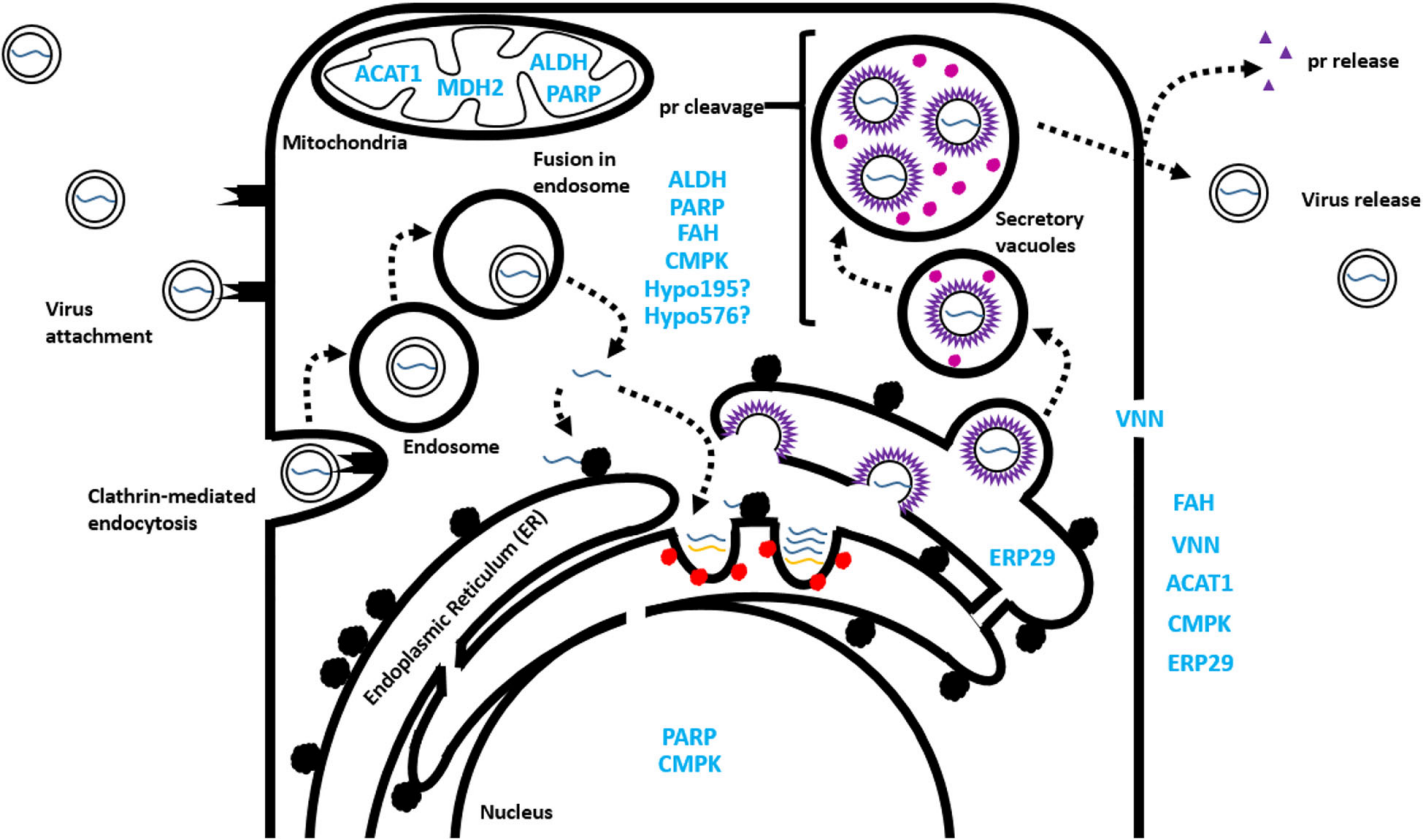
Proposed cellular location of *I. scapularis* proteins investigated in the present study. Expression of FAH, ERP29, ALDH, VNN, MDH2, PARP, CMPK, ACAT1, Hypo195 and Hypo576 (*light blue*) is shown based on studies of orthologous proteins in *H. sapiens* (excluding Hypo195 and 576). *Black* circles, ribosomes; *dark blue* lines, LGTV positive strand; *orange* lines, LGTV negative strand; *red* circles, viral proteins associated with replication complexes on the endoplasmic reticulum; *purple* particles, immature LGTV prior to cleavage of the pr peptide; *magenta* circles, furin protease produced by the host cell and responsible for cleavage of the pr peptide from the Membrane protein, producing mature LGTV; extracellular *purple* circles, cleaved pr peptide; extracellular *black* smooth particles, mature LGTV. Protein localization was based on COMPARTMENTS [33] predictions using data from *H. sapiens*

*In vitro* studies revealed no significant effect (*P* ≤ 0.05) on the viability of LGTV-infected ISE6 cells when dsRNA for the ten genes of interest was introduced either pre- or post-infection (Fig. 2a and Additional file 1: Figure S3, Table S5). dsRNA-mediated knockdown of transcripts for VNN, ACAT1, and Hypo576 revealed a decrease in relative levels (*P* ≤ 0.05) of the LGTV negative strand as compared to the negative pGEM control (Fig. 2b and Additional file 1: Table S5). Plaque assays showed that pre-treatment of ISE6 cells with dsRNA for all 10 genes of interest prior to LGTV infection resulted in a decrease (*P* ≤ 0.01) in the amount of infectious LGTV released from the cells (Fig. 2c and Additional file 1: Table S5), while treatment of ISE6 cells with dsRNA for all but one gene (PARP), following LGTV infection resulted in a decrease in amount (*P* ≤ 0.05) of infectious virus released from cells (Fig. 2d and Additional file 1: Table S5). The results of RNAi and LGTV infection studies (pre-treatment and post-treatment of cells with dsRNA) are summarized in Table 2. The location of expression of the 10 ISE6 proteins evaluated in this study was predicted using the program COMPARTMENTS and was based on homology to proteins from *H. sapiens* [33] (Fig. 3).

**Table 2:** Results of RNAi knockdown of *I. scapularis* transcripts on LGTV infection in ISE6 cells

Tick Protein	VectorBase accession ID	Effect of treatment on ISE6 protein expression^a^		Effect of transcript knockdown on LGTV negative strand replication	Effect of transcript knockdown on release of infectious LGTV
		LGTV^b^	UV-LGTV^b^		
Fumarylacetoacetase (FAH)	ISCW020196	Up	Up	No change	Decreased
Endoplasmic reticulum protein 29 (ERP29)	ISCW018425	Up	Up	No change	Decreased
Aldehyde dehydrogenase (ALDH)	ISCW015982	Up	Up	No change	Decreased
Carbon-nitrogen hydrolase/pantetheine hydrolase/vanin-like (VNN)	ISCW004822	Up	NC	Decreased	Decreased
Malate dehydrogenase (MDH2)	ISCW003528	Up	NC	No change	Decreased
Poly [ADP-ribose] polymerase (PARP)	ISCW019519	Up	NC	No change	No change
Cytidine/uridine monophosphate kinase (CMPK)	ISCW012446	Up	Up	No change	Decreased
Acetyl-CoA C-acetyltransferase (ACAT1)	ISCW016117	Up	Up	Decreased	Decreased
Hypothetical protein (Hypo195)	ISCW011195	Up	NC	No change	Decreased
Hypothetical protein (Hypo576)	ISCW020576	Up	Up	Decreased	Decreased

^a^Protein expression following LGTV and/or UV-LGTV treatment of ISE6 cells compared to mock treatment [4]. Up = increased expression and NC = no statistically-significant change in expression

^b^LGTV = LGTV infection; UV-LGTV = UV inactivated LGTV treatment

## Discussion

**Fig. 4:**
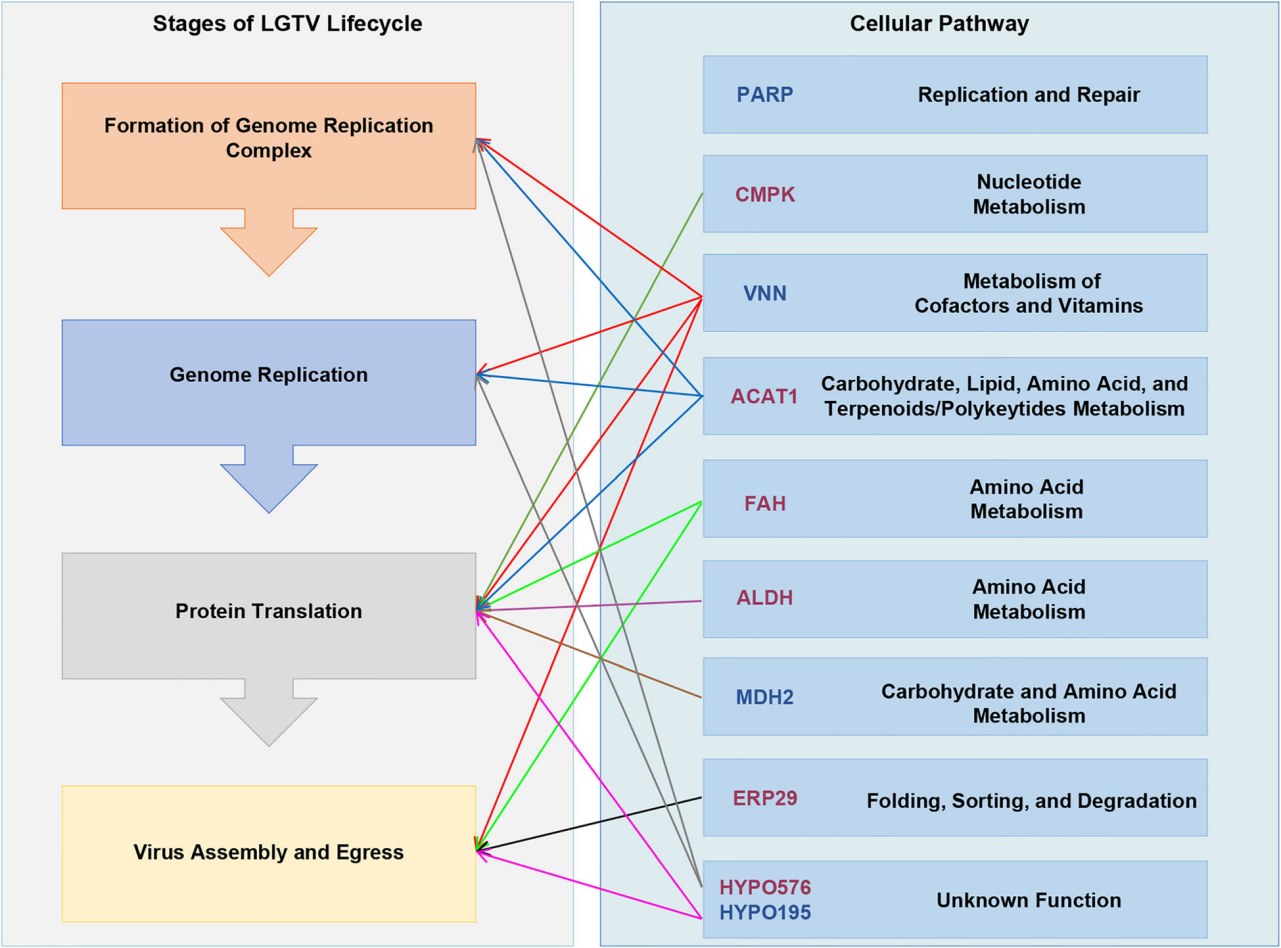
Cellular pathways of *I. scapularis* proteins and possible roles in the LGTV life-cycle inferred from RNAi data. VNN, MDH2, PARP, and Hypo195 exhibited increased expression following LGTV infection only (*blue* text). FAH, ERP29, ALDH, CMPK, ACAT1, and Hypo576 (*magenta* text) exhibited increased expression following LGTV infection and/or UV-LGTV treatment [4]. CMPK: *orange* arrow, VNN: *red* arrows, ACAT1: *blue* arrows, FAH: *green* arrows, ALDH: *purple* arrow, MDH2: *brown* arrow, ERP29: *black* arrow, Hypo576: *gray* arrows, and Hypo195: *pink* arrows. Cellular pathways were inferred based on the classification of ISE6 proteins by KEGG PATHWAY analysis and RNAi data (pre-treatment and post-treatment with dsRNA; Fig. 2b-d; Table 2)

We report the first RNAi-based study to evaluate the role of ten *I. scapularis* proteins (FAH, ERP29, ALDH, VNN, MDH2, PARP, CMPK, ACAT1, Hypo195, and Hypo576) in the LGTV life-cycle. Our results suggest that all ten proteins are involved in one or more aspects of the virus life-cycle in ISE6 cells (Fig. 2). Figure 4 shows the potential role(s) of these proteins in the LGTV life-cycle, predicted based on homology to proteins from *H. sapiens* and RNAi results*.* Here we used PCR and the LGTV negative strand template (the LGTV positive strand is copied from the negative strand in replication complexes on the endoplasmic reticulum; Fig. 3) to assess viral genome replication and plaque assays to quantify infectious virus particles released from cells. LGTV infection was reduced in the case of all ten targets when dsRNA was introduced prior to LGTV infection. Introduction of dsRNA following LGTV infection reduced viral titer for all targets except PARP, and this exception could reflect modest RNAi impact on PARP levels. Reduction in transcript levels ranged from 50 to 80% across the ten targets as compared to the control. This range is comparable to those achieved in other RNAi studies using *I. scapularis* cell lines which report reductions in transcript levels from 9 to 40% [9] and 7–100% [7] in IDE8, and from 5 to 80% in ISE6 cells [26]. Our data suggest that the RNAi effect persists in ISE6 cells for at least 60 h post-transfection, although studies to evaluate impact at the protein level are recognized as a critical next step.

Our previous investigations revealed an average replication time for LGTV in ISE6 cells of 12 h with peak release of infectious LGTV at 36 hpi [4]. Here, we pre-treated cells with dsRNA for 60 h (hereafter referred to as “pre-treatment with dsRNA”) and subsequently measured infectious LGTV particles at 16 hpi (Fig. 2c). This time-point was selected in an attempt to capture the first population of infectious virus particles released from cells. For comparative purposes, we also assessed infectious LGTV release from ISE6 cells exposed to LGTV and subsequently transfected with dsRNA for 60 h (post-treatment with dsRNA; Fig. 2d). The latter measurement may reflect several rounds of virus infection and in the case of FAH, ERP29, ALDH, MDH2, PARP, and Hypo576, may explain our observation of higher viral titers in comparison to the results for pre-treated cells.

The proposed cellular location and function of the ten *I. scapularis* proteins investigated in this study, and the predicted role(s) of these proteins in the LGTV life-cycle is depicted in Figs. 3 and 4. The knockdown of transcripts for VNN, ACAT1, and Hypo576 was associated with a decrease in both LGTV genome replication and release of infectious virus from cells (Fig. 2b-d). Further studies are required to dissect the contributions of these proteins to the LGTV infection process and determine whether the observed reduction in release of LGTV reflects the disruption of processes pre- and/or post-replication of the viral genome. The knockdown of transcripts for FAH, ERP29, ALDH, MDH2, PARP, CMPK, and Hypo195 was associated with a decrease in infectious LGTV only, suggesting that these proteins may be involved in processes independent of viral genome replication (i.e. viral protein synthesis, packaging, egress, and release).

VNN is a glycosylphosphatidylinositol (GPI)-anchored protein [34] found at the surface of many different vertebrate cells [35] and may also be secreted [33]. GPI proteins perform diverse cellular functions, including lipid remodeling, which occurs during the replication of flaviviruses. KEGG pathway analyses suggest that VNN may function in pantothenate/coenzyme-A (CoA) biosynthesis [36], which is critical for lipid metabolism. This protein family is worthy of further investigation in *I. scapularis* and other TBF vectors as antiviral targets. ACAT1 may be expressed in the mitochondria and potentially secreted [33]. This enzyme likely utilizes CoA metabolites during metabolism of fatty acids (lipids) and amino acids [37] and may be linked to formation of the lipid-based replication complex in *I. scapularis* cells through provision of lipids. Small molecules that target enzymes associated with fatty acid and lipid metabolism, such as fatty-acid synthase [10, 11, 13, 38], have been associated with a reduction in mosquito-borne flavivirus infection. However, the possible interaction of ACAT1 with multiple binding partners (Table 1) deserves further investigation as enzymatic promiscuity could impact the potential of this molecule as a target for development of antiviral compounds and transmission blocking vaccines.

ALDH is likely expressed in the mitochondria and cytosol [33] and associated with amino acid metabolism [37] in tick cells. Members of this enzyme class complete the conversion of delta-1-pyrroline-5-carboxylate (P5C) to glutamate, a necessary step linking the urea and tricarboxylic acid cycles with glutaminolysis. In tick cells, the presumably mitochondrial MDH2 [33] may function in pyruvate metabolism [37] and as a rate-limiting enzyme in the citrate cycle. Both ALDH and MDH2 were assigned to the biological processes “ribonucleoprotein/ribosomal/translation/protein metabolic function” (Additional file 1; Table S3) and RNAi data support involvement of these metabolic pathways in viral production in tick cells, possibly *via* effects on the translation of virus or host RNA. ALDH and MDH2 may represent targets for antivirals and anti-tick vaccines although their potential interaction with multiple binding partners (Table 1) and involvement in multiple cellular processes requires further evaluation. CMPK may be expressed in the cytosol and nucleus [33], and may be involved in nucleotide metabolism in *I. scapularis* [37]. We speculate that a reduction in this enzyme may reduce nucleotides for DNA/RNA synthesis, and suppress virus production. Suppression of pyrimidine biosynthesis was associated with reduced DENV infection [39] and small molecules that inhibit other enzymes involved in nucleotide biosynthesis reduced arboviruses in cell culture [13, 40, 41]. Inhibition of CMPK may reduce TBF infection but would likely have broad impacts on cellular processes. ALDH, MDH2 and CMPK exhibited increased expression in human hepatoma 7.5 cells (HUH7.5) infected with Hepatitis C virus (HCV) [42] and FAH was decreased in HCV-associated carcinoma tissue [43]. Products that disrupt host proteins involved in enzymatic pathways commonly manipulated by flaviviruses, may provide broad anti-viral effect.

Analyses suggest FAH is a secreted and cytosolic protein that may perform amino acid metabolism [33, 37] in *I. scapularis*. Disruption of amino acid synthesis could affect the production of virus or host proteins at one or more points in the virus life-cycle. ERP29 is likely associated with the endoplasmic reticulum (ER) and also secreted [33]), and may be involved in protein processing and ER associated degradation [37] in the tick. ERP29 showed increased expression in mouse brain tissue following infection with the Japanese Encephalitis virus (JEV) [44]. Studies of polyomavirus-infected cells also suggest a role for ERP29 in viral binding and release from the ER lumen [45–47]. Additional studies are required to investigate the role of ERP in LGTV infection and evaluate the potential of this target.

PARP is likely expressed in the cytosol, mitochondria, and nucleus [33] and may be involved in DNA replication and repair [37] in the tick. This protein has a role in pro-apoptotic signaling and is activated by oxidative stress [48]. The HCV non-structural protein 5A (NS5A) can create oxidative stress, leading to activation of PARP [49]. JEV and DENV also induced cleavage of PARP1, resulting in a variety of pro-apoptotic responses [50]. The increased expression of the ISE6 proteins observed on infection with LGTV may reflect a generalized cellular response or metabolic processes and products used by the virus [4]. Flaviviruses are thought to exploit the cellular stress response to aid replication [51] and oxidative stress is thought to aid the replication of positive-strand RNA viruses [52]. Further, it has been proposed that the balance between antioxidant responses maintains an anti-apoptotic environment during flavivirus infection of the cell [53, 54], presumably facilitating virus replication and transmission.

LGTV concentration has been linked to efficiency of establishment of infection in *I. scapularis* larvae [27]. In the present study, transcript knockdown produced a modest reduction in LGTV replication and genome replication. It is recognized that a small reduction in amount of infectious virus can profoundly affect transmission; further functional analyses might investigate transcript knockdown over time and it will be necessary to establish that a reduction in protein level or impairment of enzymatic activity *in vivo* translates to a meaningful reduction in virus transmission. Recapitulation of such studies in the natural vectors of LGTV will also be important as flavivirus replication may differ among natural vector and non-vector cell types [55]. Our predictions regarding cellular compartments associated with protein expression were made based on homology to proteins from *H. sapiens* and studies are needed to determine the spatio-temporal expression of tick proteins. Transcripts for MDH2 were identified in the salivary glands of blood-fed *I. scapularis* nymphs [56] and both MDH2 and Hypo195 were identified in the synganglia of *I. scapularis* [6] suggesting potential roles in neurological processes. Functional studies would be of particular value in the case of Hypo195 and Hypo576. Orthologs of Hypo195 and Hypo576 have not been identified in *H. sapiens* (Table 1) and these proteins may represent targets for development of vector-specific products to control flavivirus transmission.

## Conclusions

Our work provides for investigations of orthologous protein targets in transmission of more virulent TBFs, including POWV and TBEV. Theoretically, small molecules that disrupt one or more tick proteins could be used to limit virus transmission from the tick to mammalian reservoirs and intermediate hosts. Small molecule inhibitors of FAH [57, 58] and PARP [59, 60] are known but their potential to regulate flavivirus infection in arthropods has not been investigated. In addition, there is precedent for development of transmission blocking vaccines against TBFs. The outer surface protein A (OspA) of the *Borrelia burgdorferi* bacterium is the basis for a Lyme disease (LD) vaccine and has been deployed in the *Peromyscus leucopus* (white-footed mouse) reservoir [61, 62]. This vaccine, delivered *via* oral bait, offers one strategy to reduce circulation of *B. burgdorferi* in the reservoir, and subsequent transmission to the tick vector. Functional studies described here highlight proteins associated with pathogenesis of TBFs and are a necessary precursor to anti-tick vaccine development [63]. Further functional studies will reveal the potential of these proteins as targets for development of new strategies to prevent TBF infections.

## Additional file

Additional file 1: Figure S1. Summary of the process employed to select *I. scapularis* genes for RNAi knockdown experiments. ^Δ^ ISE6 proteins from the differential proteomic analysis at 36 hpi were analyzed. Proteins were selected based on (1) increased expression level, (2) strength of proteomic support (minimum 2 peptides identified from LC-MS-MS per protein) from proteins identified in Grabowski et al. [4], and (3) orthology to vertebrate/invertebrate proteins; * orthologous proteins identified in published proteomic studies [4–6, 8]. LGTV denotes proteins that exhibited increased expression following LGTV infection and LGTV & UV-LGTV denotes proteins that exhibited increased expression following both LGTV infection and UV-LGTV treatment. + proteins that exhibited increased expression following LGTV infection as compared to UV-LGTV treatment. FAH, fumarylacetoacetase; ERP29, endoplasmic reticulum protein 29; ALDH, 1-pyrroline-5-carboxylate dehydrogenase; VNN, pantetheine hydrolase; MDH2, malate dehydrogenase; PARP, poly [ADP-ribose] polymerase; CMPK, UMP-CMP kinase; ACAT1, acetyl-CoA acetyltransferase; Hypo195, hypothetical protein; Hypo576. The prefix “ISCW” denotes VectorBase accession IDs. **Figure S2** Effect of pGEM dsRNA concentrations on ISE6 cell viability following transfection for 60 h. X-tremeGENE (Xtr) transfection reagent was used to optimize pGEM dsRNA (RNAi negative control) concentrations in ISE6 cells at 60 h post transfection. Cell viability readings were compared to the Xtr + OptiMEM (Opti) control (*gray* bar). Red boxes indicate increased or no significant decrease in ISE6 cell viability. RLU_560,590_, relative light units 560 nm excitation and 590 nm emission. Error bars represent SEM. Statistical analysis was performed using an unpaired t-test between Xtr + Opti control and each pGEM dsRNA concentration. **p* value ≤ 0.05 and ***p* value ≤ 0.01. Results represent 3 technical replicates and 1 biological replicate (multiple biological replicates completed with 10 ng concentration). **Figure S3** Effect of transfection with dsRNA on ISE6 cell viability. FAH, fumarylacetoacetase; ERP29, endoplasmic reticulum protein 29; ALDH, 1-pyrroline-5-carboxylate dehydrogenase; VNN, pantetheine hydrolase; MDH2, malate dehydrogenase; PARP, poly [ADP-ribose] polymerase; CMPK, UMP-CMP kinase; ACAT1, acetyl-CoA acetyltransferase; Hypo195, hypothetical protein; Hypo576, hypothetical protein; pGEM, pGEM plasmid (negative control; *light gray* bars); LGTV 3UTR, 3’ UTR of LGTV TP21 strain (positive control; *dark gray* bars), RLU_560,590_, relative light units 560 nm excitation and 590 nm emission. ISE6 cell viability following transfection with 10ng dsRNA for 60 h normalized to the negative control pGEM dsRNA. Results represent 2–5 technical replicates and 3 biological replicates. Error bars represent SEM and unpaired t-tests for comparison of cell viability of the negative pGEM control versus each gene of interest. **Table S1** T7-tagged primers used to amplify cDNA and synthesize dsRNA. **Table S2** Primers used to amplify cDNA for *I. scapularis* genes of interest by RT-qPCR. **Table S3** Enrichment/cluster analysis of ISE6 proteins that exhibited increased expression following LGTV and UV-LGTV treatment. ISE6 proteins with increased expression following LGTV infection and/or UV-LGTV treatment from [4] were searched via DAVID enrichment analysis. For each cluster, the *P* value represents a modified Fisher Exact *P* value, and EASE score implemented in DAVID gene enrichment and functional annotation analysis. Enrichment (E) score of ≥ 1.3 is equal to *P* value of ≤ 0.05. **Table S4** Nucleotide similarity of RT-PCR products amplified from *I. scapularis* and ISE6 cells and IscaW1 gene models. **Table S5** Summary of statistically significant values corresponding to figures. (DOCX 329 kb)

## Abbreviations

ACAT1: Acetyl-CoA acetyltransferase
ALDH: Aldehyde dehydrogenase
ATCC: American Tissue Culture Collection
cDNA: Complementary DNA
CMPK: Cytidine/uridine monophosphate kinase
DENV: Dengue virus
dsRNA: Double stranded RNA
ER: Endoplasmic reticulum
ERP29: Endoplasmic reticulum protein 29
FAH: Fumarylacetoacetase
GO: Gene ontology
HCV: Hepatitis C virus
HUH: Human hepatoma cell line
Hypo195/576: Hypothetical proteins 195/576
IDE8/ISE6: *Ixodes scapularis* cell line 8/6
IFA: Immunofluorescence assay
JEV: Japanese encephalitis
KEGG: Kyoto Encyclopedia of Genes and Genomes
LD: Lyme disease
LGTV: Langat virus
MDH2: Malate dehydrogenase 2
MOI: Multiplicity of infection
Opti: OptiMEM
PARP: Poly [ADP-ribose] polymerase
RNAi: RNA interference
RT-qPCR: reverse transcriptase-quantitative polymerase chain reaction
SEM: Standard error of the mean
TBF: Tick-borne flavivirus
VNN: Carbon nitrogen hydrolase/vanin-like
Xtr: X-tremeGENE transfection reagent
