# Supplementary material for: RNAi reveals proteins for metabolism and protein processing associated with Langat virus infection in Ixodes scapularis (black-legged tick) ISE6 cells
Source: Parasit Vectors. 2017 Jan 13;10:24. doi: 10.1186/s13071-016-1944-0 (PMC5237174; doi:10.1186/s13071-016-1944-0)
Supplement: Additional file 1: Figure S1. — Summary of the process employed to select I. scapularis genes for RNAi knockdown experiments. Δ ISE6 proteins from the differential proteomic analysis at 36 hpi were analyzed. Proteins were selected based on (1) increased expression level, (2) strength of proteomic support (minimum 2 peptides identified from LC-MS-MS per protein) from proteins identified in Grabowski et al. [4], and (3) orthology to vertebrate/invertebrate proteins; * orthologous proteins identified in published proteomic studies [4–6, 8]. LGTV denotes proteins that exhibited increased expression following LGTV infection and LGTV & UV-LGTV denotes proteins that exhibited increased expression following both LGTV infection and UV-LGTV treatment. + proteins that exhibited increased expression following LGTV infection as compared to UV-LGTV treatment. FAH, fumarylacetoacetase; ERP29, endoplasmic reticulum protein 29; ALDH, 1-pyrroline-5-carboxylate dehydrogenase; VNN, pantetheine hydrolase; MDH2, malate dehydrogenase; PARP, poly [ADP-ribose] polymerase; CMPK, UMP-CMP kinase; ACAT1, acetyl-CoA acetyltransferase; Hypo195, hypothetical protein; Hypo576. The prefix “ISCW” denotes VectorBase accession IDs. Figure S2 Effect of pGEM dsRNA concentrations on ISE6 cell viability following transfection for 60 h. X-tremeGENE (Xtr) transfection reagent was used to optimize pGEM dsRNA (RNAi negative control) concentrations in ISE6 cells at 60 h post transfection. Cell viability readings were compared to the Xtr + OptiMEM (Opti) control (gray bar). Red boxes indicate increased or no significant decrease in ISE6 cell viability. RLU560,590, relative light units 560 nm excitation and 590 nm emission. Error bars represent SEM. Statistical analysis was performed using an unpaired t-test between Xtr + Opti control and each pGEM dsRNA concentration. *p value ≤ 0.05 and **p value ≤ 0.01. Results represent 3 technical replicates and 1 biological replicate (multiple biological replicates completed with 10 ng concentration). Fig [file 13071_2016_1944_MOESM1_ESM.docx]

**Additional File 1.**


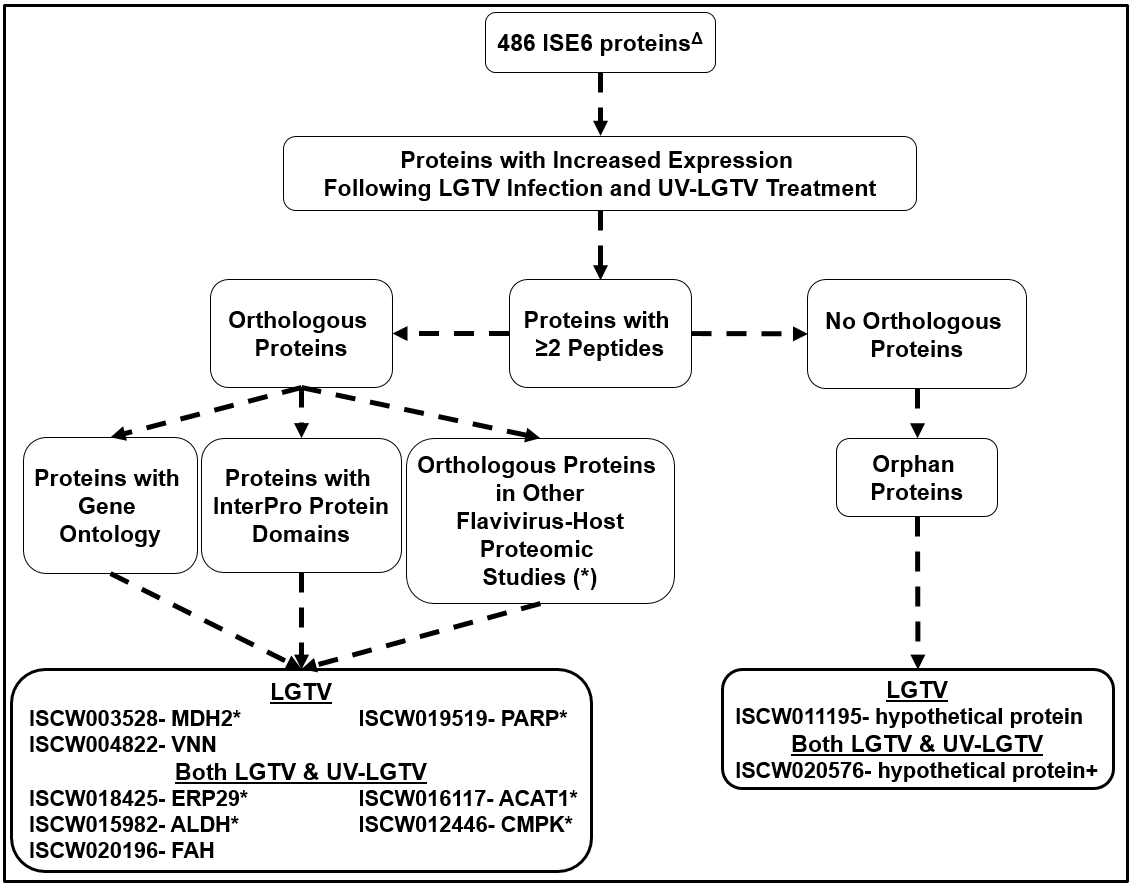


**Fig. S1. Summary of process employed to select *I. scapularis* genes for RNAi knockdown experiments.** ^Δ^ ISE6 proteins from the differential proteomic analysis at 36 hpi were searched. Proteins were selected based on (1) increased expression level, (2), strength of proteomic support (minimum 2 peptides from LC-MS-MS identified to a protein) from proteins identified in Grabowski et al [[1](#_ENREF_1)], and (3) orthology to vertebrate/invertebrate proteins; * orthologous proteins identified in published proteomic studies of flavivirus-host interactions [[2-5](#_ENREF_2)]. LGTV denotes proteins found with increased expression following LGTV infection and both LGTV & UV-LGTV denotes proteins with increased expression following both LGTV infection and UV-LGTV treatment. + proteins identified with greater expression with LGTV infection than with UV-LGTV treatment. FAH, fumarylacetoacetase; ERP29, endoplasmic reticulum protein 29; ALDH, 1-pyrroline-5-carboxylate dehydrogenase; VNN, pantetheine hydrolase; MDH2, malate dehydrogenase; PARP, poly [ADP-ribose] polymerase; CMPK, UMP-CMP kinase; ACAT1, acetyl-CoA acetyltransferase; Hypo195, hypothetical protein; Hypo576. The prefix “ISCW” denotes VectorBase accession IDs.


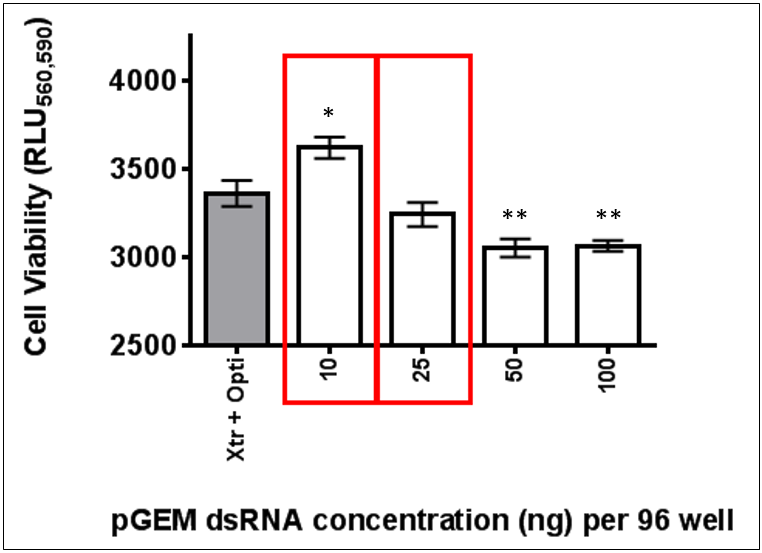


**Fig. S2.** **Effect of pGEM dsRNA concentrations on ISE6 cell viability following transfection for 60 hours.** X-tremeGENE (Xtr) transfection reagent was used to optimize pGEM dsRNA (RNAi negative control) concentrations in ISE6 cells at 60 hours post transfection. Cell viability readings were compared to the Xtr + OptiMEM (Opti) control (gray bar). Red boxes indicate increased/no decrease in ISE6 cell viability. RLU_560,590_, relative light units (fluorescent reading on Molecular Devices SpectraMax M5 plate reader with 560 nm excitation and 590 nm emission coupled with SoftMax Pro v4.8 software). Error bars represent SEM. Statistical analysis was performed using an unpaired t-test between Xtr + Opti control and each pGEM dsRNA concentration. Asterisks denote: *p value ≤ 0.05 and **p value ≤ 0.01. Results represent 3 technical replicates and 1 biological replicate (multiple biological replicates completed with 10 ng concentration).

**
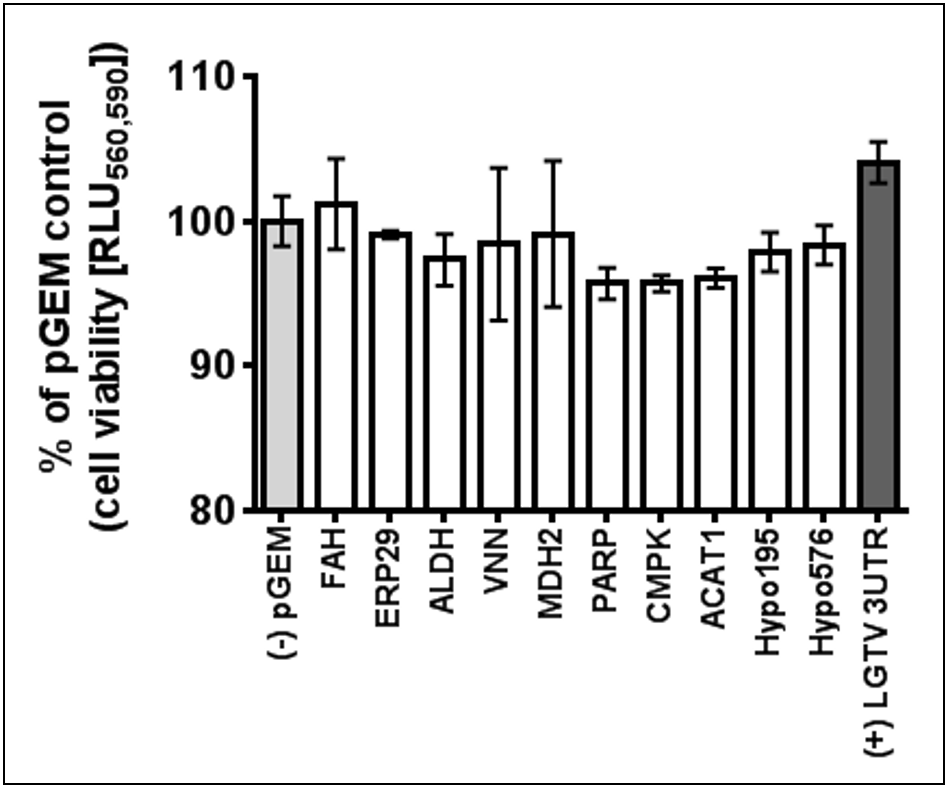
**

**Fig. S3. Effect of transfection with dsRNA for genes of interest on ISE6 cell viability.** FAH, fumarylacetoacetase; ERP29, endoplasmic reticulum protein 29; ALDH, 1-pyrroline-5-carboxylate dehydrogenase; VNN, pantetheine hydrolase; MDH2, malate dehydrogenase; PARP, poly [ADP-ribose] polymerase; CMPK, UMP-CMP kinase; ACAT1, acetyl-CoA acetyltransferase; Hypo195, hypothetical protein; Hypo576, hypothetical protein; pGEM, pGEM plasmid (negative control; light gray bars); LGTV 3UTR, 3’ UTR of LGTV TP21 strain (positive control; dark gray bars), RLU_560,590_, relative light units 560 nm excitation and 590 nm emission. ISE6 cell viability following transfection with 10ng dsRNA for 60 hours normalized to the negative control pGEM dsRNA. Results represent 2-5 technical replicates and 3 biological replicates. Error bars represent SEM and unpaired t-tests compared cell viability of the negative pGEM control versus each gene of interest.

**Table S1. T7-tagged primers used to amplify cDNA and synthesize dsRNA.**

| **Primer Name** | **Primer Sequence** | **Expected size of amplified cDNA (bp)** |
| --- | --- | --- |
| F T7-FAH | 5’-TAATACGACTCACTATAGGGTTCCAATGTCCCAGGCTA-3’ | 551 |
| R T7-FAH | 5’-TAATACGACTCACTATAGGGCCTTGTGAAGGAGGTAGGGC-3’ |  |
| F T7-ERP29 | 5’-TAATACGACTCACTATAGGGCGAAGTTCAAAGTGACGCTGG-3’ | 553 |
| R T7-ERP29 | 5’-TAATACGACTCACTATAGGGCTGGTGATCTTTCCGTTGCG-3’ |  |
| F T7-ALDH | 5’-TAATACGACTCACTATAGGGTCAGCCCATCACAACAGACC-3’ | 534 |
| R T7-ALDH | 5’-TAATACGACTCACTATAGGGTTGTCCGGCACGTAGACAC-3’ |  |
| F T7-VNN | 5’-TAATACGACTCACTATAGGGTACCACGGTGACGAGATTGC-3’ | 607 |
| R T7-VNN | 5’-TAATACGACTCACTATAGGGTATGTGAGCTCCGCTTGAGG-3’ |  |
| F T7-MDH2 | 5’-TAATACGACTCACTATAGGGCTGCGGACAAATCGCCAAAG-3’ | 310 |
| R T7-MDH2 | 5’-TAATACGACTCACTATAGGGTGAAGCCCTTGACTTGAGGC-3’ |  |
| F T7-PARP | 5’-TAATACGACTCACTATAGGGCAAGGATCTTCCGCTGGAT-3’ | 310 |
| R T7-PARP | 5’-TAATACGACTCACTATAGGGCGAAGACTCACGTCTCCACC-3’ |  |
| F T7-CMPK | 5’-TAATACGACTCACTATAGGGTACAAGCACCTGTCGGCG-3’ | 349 |
| R T7-CMPK | 5’-TAATACGACTCACTATAGGGTGAGGCTCTCCATGTTGTCG-3’ |  |
| F T7-ACAT1 | 5’-TAATACGACTCACTATAGGGCGTCTACGATGGCCTGACTG-3’ | 392 |
| R T7-ACAT1 | 5’-TAATACGACTCACTATAGGGTTCGAGCAATGGGCTTCACT-3’ |  |
| F T7-Hypo195 | 5’-TAATACGACTCACTATAGGGCAACAAACTCGGCGGCTTC-3’ | 300 |
| R T7-Hypo195 | 5’-TAATACGACTCACTATAGGGCACCCTTCAGGAAGTTGCTC-3’ |  |
| F T7-Hypo576 | 5’-TAATACGACTCACTATAGGGAGCCCAACTGCATCTACC-3’ | 483 |
| R T7-Hypo576 | 5’-TAATACGACTCACTATAGGGTGAGCCTTGATGCGGTCTAC-3’ |  |
| F T7-pGEM | 5’-TAATACGACTCACTATAGGGGGTATCAGCTCACTCAAAGG-3’ | 362 |
| R T7-pGEM | 5’-TAATACGACTCACTATAGGGGAACGACCTACACCGAACT-3’ |  |
| F T7-LGTV 3UTR^1^ | 5’-TAATACGACTCACTATAGGGCCAGACACAAGGAGTCCAA-3’ | 510 |
| R T7-LGTV 3UTR^1^ | 5’-TAATACGACTCACTATAGGGATGGTGGCTCAGGGAGAAC-3’ |  |

*FAH, fumarylacetoacetase; ERP29, endoplasmic reticulum protein 29; ALDH, 1-pyrroline-5-carboxylate dehydrogenase; VNN, pantetheine hydrolase; MDH2, malate dehydrogenase; PARP, poly [ADP-ribose] polymerase; CMPK, UMP-CMP kinase; ACAT1, acetyl-CoA acetyltransferase; Hypo195, hypothetical protein; Hypo576, hypothetical protein; pGEM, pGEM plasmid (negative control); LGTV 3UTR, 3’ UTR of LGTV TP21 strain (positive control).

*T7 promoter sequence tag shown underlined.

*F, forward primer; R, reverse primer.

^1^GenBank accession EU790644.1 identified through Virus Pathogen Resource (viprbrc.org/brc).

**Table S2. Primers used to amplify cDNA for *I. scapularis* genes of interest by RT-qPCR.**

| **Primer Name** | **Primer Sequence** |
| --- | --- |
| F FAH | 5’-TCACGTTCGAGAATCTGCCG-3’ |
| R FAH | 5’-GTAGTCTCCTATGGCCACGC-3’ |
| F ERP29 | 5’-TCAAAGCGGTCTCACGCTAC-3’ |
| R ERP29 | 5’-GTACGCTGCCCTTTGTGGTG-3’ |
| F ALDH | 5’-TTGGAGGCAAGGAGATCAGG-3’ |
| R ALDH | 5’-CAGGCTGGTGTTTATCGCCT-3’ |
| F VNN | 5’-CTACAACACCAACGTGGCCT-3’ |
| R VNN | 5’-AAGGGCTCCACAAACAGGTG-3’ |
| F MDH2 | 5’-GGTGTTCAAGAAGCGGGGT-3’ |
| R MDH2 | 5’-ACGTTCACTGTGGCTGGGTC-3’ |
| F PARP | 5’-CTCGACGGAGTATGCCAAGT-3’ |
| R PARP | 5’-CATCTTGGAGAAGGCCCCAT-3’ |
| F CMPK | 5’-GGCCTCTTGACCATGTCTGG-3’ |
| R CMPK | 5’-AATCTTCTGACACTGGGTGCC-3’ |
| F ACAT1 | 5’-CAGGAGTTCTTTGGCTGCTG-3’ |
| R ACAT1 | 5’-TGAACACCTCCTGGACATCG-3’ |
| F Hypo195 | 5’-CACCTCGACAAACTGAGCAC-3’ |
| R Hypo195 | 5’-CGTCCGTGCATAACTGGCAA-3’ |
| F Hypo576 | 5’-TGGCTGAACACGGACATCAAG-3’ |
| R Hypo576 | 5’-TCGGGCTCAACAGGGGAC-3’ |
| F β-actin | 5’-GCCGGGACCTTACAGACTATC-3’ |
| R β-actin | 5’-CACGGACAATTTCACGCTCG-3’ |
| F β-tubulin | 5’-ACCTTCATTGGCAACAGCAC-3’ |
| R β-tubulin | 5’-CCCTCTCCGGTGTACCAGT-3’ |
| F LGTV negative strand^a^ | 5’-GTCTCCGGTTGCAGGACTGT-3’ |
| R LGTV negative strand^a^ | 5’-CTCGGTCAGTAGGATGGTGTTG-3’ |

*FAH, fumarylacetoacetase; ERP29, endoplasmic reticulum protein 29; ALDH, 1-pyrroline-5-carboxylate dehydrogenase; VNN, pantetheine hydrolase; MDH2, malate dehydrogenase; PARP, poly [ADP-ribose] polymerase; CMPK, UMP-CMP kinase; ACAT1, acetyl-CoA acetyltransferase; Hypo195, hypothetical protein; Hypo576, hypothetical protein; β-actin, beta-actin internal control; β-tubulin, beta-tubulin internal control; LGTV negative strand, LGTV TP21 strain.

*F, forward primer; R, reverse primer.

^a^Genebank accession no. AF253419; primers derived from Mitzel et al [[6](#_ENREF_6)].

**Table S3. Enrichment/cluster analysis of ISE6 proteins that exhibited increased expression following LGTV and UV-LGTV treatment.** ISE6 proteins with increased expression following LGTV infection and/or UV-LGTV treatment from the differential proteomic analysis [[1](#_ENREF_1)] were searched via DAVID enrichment analysis. For each cluster, the P value represents a modified Fisher Exact P value, an EASE score implemented in DAVID gene enrichment and functional annotation analysis. Enrichment (E) score of ≥ 1.3 is equal to a ≤ 0.05 P value; represents the EASE scores of all members of the cluster.

| **Cluster Function Highlighted** | **VectorBase Accession IDs** |
| --- | --- |
| **LGTV** | |
| Translation/ribosomal function/protein metabolic processing  (E: 1.43 / P: ≤0.049) | ISCW023068, ISCW010723, ISCW014576, ISCW017347, ISCW019519, ISCW003528 |
| **UV-LGTV** | |
| Cellular protein transport  (E: 1.3 / P: ≤0.027) | ISCW009326, ISCW017299, ISCW021969 |
| **LGTV & UV-LGTV** | |
| Nitrogen metabolic processing  (E: 1.71 / P: ≤0.048) | ISCW000393, ISCW003100, ISCW003709, ISCW012446, ISCW015982, ISCW020196, ISCW021269, ISCW007139, ISCW016117 |
| Nitrogen/amine/amino acid metabolic processing  (E: 1.61 / P: ≤0.042) | ISCW000393, ISCW015982, ISCW020196, ISCW012446, ISCW021269 |
| **LGTV, UV-LGTV, and LGTV & UV-LGTV** | |
| Ribonucleoprotein/ribosomal/translation/protein metabolic function (E: 1.94 / P: ≤0.047) | ISCW023068, ISCW008104, ISCW010723, ISCW017299, ISCW017347, ISCW021269, ISCW021749, ISCW023883, ISCW000393, ISCW003528, ISCW003709, ISCW011597, ISCW012446, ISCW013384, ISCW014576, ISCW015982, ISCW019519, ISCW020196, ISCW007139, ISCW003100 |
| Genetic/transcriptional control  (E: 1.53 / P: ≤0.034) | ISCW001860, ISCW010309, ISCW019664 |

*Red, six proteins selected for RNAi analyses in this study. “LGTV only” denotes proteins with increased expression following LGTV infection, “UV-LGTV only” denotes proteins with increased expression following UV-LGTV treatment, “LGTV & UV-LGTV” denotes proteins with increased expression following both LGTV infection and UV-LGTV treatment, and “LGTV only, UV-LGTV only, and LGTV & UV-LGTV” denotes all proteins identified in the previous three datasets. The prefix “ISCW” denotes VectorBase accession IDs.

**Table S4. Similarity between RT-PCR products amplified from *I. scapularis* and ISE6 cells and IscaW1 gene models.**

| **Protein Name** | **Sequenced cDNA Length (bp)** | **% Coverage: Female *I. scapularis* RT-PCR amplicon to IscaW1 gene model^1^** | **BLAST Score / Alignment Expectation Value** | **% Coverage:**  **ISE6 cell RT-PCR amplicon to IscaW1**  **gene model^a^** | **BLAST Score / Alignment Expectation Value** |
| --- | --- | --- | --- | --- | --- |
| FAH | 551 | 99% | 932 / 0.0 | 98% | 955 / 0.0 |
| ERP29 | 553 | 99% | 950 / 0.0 | 99% | 900 / 0.0 |
| ALDH | 534 | 98% | 870 / 0.0 | 98% | 876 / 0.0 |
| VNN | 607 | 97% | 965 / 0.0 | 96% | 828 / 0.0 |
| MDH2 | 310 | 99% | 488 / e^-134^ | 97% | 458 / e^-125^ |
| PARP | 310 | 96% | 416 / e^-113^ | 99% | 490 / e^-135^ |
| CMPK | 349 | 99% | 513 / e^-142^ | 99% | 488 / e^-134^ |
| ACAT1 | 392 | 98% | 452 / e^-123^ | 98% | 484 / e^-133^ |
| Hypo195 | 300 | 99% | 472 / e^-129^ | 99% | 464 / e^-127^ |
| Hypo576 | 483 | 99% | 827 / 0.0 | 100% | 852 / 0.0 |

*RNA from a single adult female *I. scapularis* and ISE6 cell population was used to create the cDNA template for PCR.

^a^% nucleotide coverage between amplicon and corresponding IscaW1 gene model.

*Wikel strain *I. scapularis* IscaW1 assembly.

**Table S5. Summary of statistically-significant values corresponding to figures.**

|  | **Protein Name** | ***t* value** | **Degrees freedom** | ***P* value** |
| --- | --- | --- | --- | --- |
| **Fig. 1^×^** | | | | |
|  | FAH | 2.935 | 6 | 0.0261 |
|  | ERP29 | 6.483 | 6 | 0.0006 |
|  | ALDH | 5.792 | 6 | 0.0012 |
|  | VNN | 6.44 | 6 | 0.0007 |
|  | MDH2 | 5.3 | 6 | 0.0018 |
|  | PARP | 2.955 | 6 | 0.0254 |
|  | CMPK | 2.97 | 6 | 0.0249 |
|  | ACAT1 | 5.517 | 6 | 0.0015 |
|  | Hypo195 | 5.107 | 6 | 0.0022 |
|  | Hypo576 | 5.562 | 6 | 0.0014 |
| **Fig. 2a^π^** | | | | |
|  | FAH | 0.3637 | 12 | 0.7224 |
|  | ERP29 | 0.08545 | 12 | 0.9333 |
|  | ALDH | 0.225 | 12 | 0.8258 |
|  | VNN | 0.7445 | 12 | 0.4709 |
|  | MDH2 | 1.346 | 12 | 0.2030 |
|  | PARP | 0.007057 | 12 | 0.9945 |
|  | CMPK | 1.102 | 12 | 0.2919 |
|  | ACAT1 | 0.5857 | 12 | 0.5690 |
|  | Hypo195 | 0.6097 | 12 | 0.5534 |
|  | Hypo576 | 0.09952 | 12 | 0.9224 |
|  | LGTV 3’UTR | 0.7994 | 12 | 0.4396 |
| **Fig. 2b^∞^** | | | | |
|  | FAH | 0.1691 | 8 | 0.8699 |
|  | ERP29 | 0.8356 | 8 | 0.4276 |
|  | ALDH | 1.682 | 8 | 0.1310 |
|  | VNN | 2.428 | 8 | 0.0413 |
|  | MDH2 | 0.5552 | 8 | 0.5939 |
|  | PARP | 1.525 | 8 | 0.1657 |
|  | CMPK | 2.291 | 8 | 0.0512 |
|  | ACAT1 | 3.023 | 8 | 0.0165 |
|  | Hypo195 | 2.173 | 8 | 0.0615 |
|  | Hypo576 | 3.133 | 8 | 0.0140 |
|  | LGTV 3’UTR | 4.981 | 8 | 0.0011 |
| **Fig. 2c^α^** | | | | |
|  | FAH | 6.03 | 9 | 0.002 |
|  | ERP29 | 6.718 | 9 | <0.0001 |
|  | ALDH | 6.026 | 9 | 0.002 |
|  | VNN | 4.937 | 9 | 0.0008 |
|  | MDH2 | 5.641 | 9 | 0.0003 |
|  | PARP | 4.223 | 9 | 0.0022 |
|  | CMPK | 5.551 | 9 | 0.0004 |
|  | ACAT1 | 5.617 | 9 | 0.0003 |
|  | Hypo195 | 6.143 | 9 | 0.0002 |
|  | Hypo576 | 6.345 | 9 | 0.0001 |
|  | LGTV 3’UTR | 11.53 | 9 | <0.0001 |
| **Fig. 2d^α^** | | | | |
|  | FAH | 2.981 | 15 | 0.0093 |
|  | ERP29 | 3.952 | 15 | 0.0013 |
|  | ALDH | 3.56 | 15 | 0.0029 |
|  | VNN | 3.985 | 15 | 0.0012 |
|  | MDH2 | 2.482 | 15 | 0.0254 |
|  | PARP | 1.941 | 15 | 0.0712 |
|  | CMPK | 4.17 | 15 | 0.0008 |
|  | ACAT1 | 4.594 | 15 | 0.0004 |
|  | Hypo195 | 4.935 | 15 | 0.0002 |
|  | Hypo576 | 3.305 | 15 | 0.0048 |
|  | LGTV 3’UTR | 9.003 | 15 | <0.0001 |
| **Fig. S2^β^** | | | | |
|  | 10 ng | 2.725 | 8 | 0.0260 |
|  | 25 ng | 1.181 | 8 | 0.2716 |
|  | 50 ng | 3.451 | 8 | 0.0087 |
|  | 100 ng | 3.729 | 8 | 0.0058 |
| **Fig. S3^π^** | | | | |
|  | FAH | 0.3458 | 6 | 0.7413 |
|  | ERP29 | 0.7722 | 6 | 0.7722 |
|  | ALDH | 0.8235 | 6 | 0.4417 |
|  | VNN | 0.3986 | 6 | 0.7040 |
|  | MDH2 | 0.2226 | 6 | 0.8312 |
|  | PARP | 1.358 | 6 | 0.2234 |
|  | CMPK | 1.37 | 6 | 0.2197 |
|  | ACAT1 | 1.252 | 6 | 0.2571 |
|  | Hypo195 | 0.6684 | 6 | 0.5287 |
|  | Hypo576 | 0.5166 | 6 | 0.6239 |
|  | LGTV 3’UTR | 1.267 | 6 | 0.2521 |

^×^Unpaired *t-*test between % mRNA values observed for the dsRNA treatment for each gene/protein of interest versus the negative (pGEM) control.

^π^Unpaired *t*-test between % cell viability (RLU_560,590_) values observed for the dsRNA treatment for each gene/protein of interest versus the negative (pGEM) control.

^∞^Unpaired *t*-test between % negative strand genome replication values observed for the dsRNA treatment for each gene/protein of interest versus the negative (pGEM) control .

^α^Unpaired *t*-test between % pfu/ml values observed for the dsRNA treatment for each gene/protein of interest versus the negative pGEM control.

^β^Unpaired *t*-test between cell viability (RLU_560,590_) values observed for the negative (Xtr + Opti) control versus pGEM dsRNA concentrations.

**REFERENCES**

1. Grabowski JM, Perera R, Roumani AM, Hedrick VE, Inerowicz HD, Hill CA, et al. Changes in the Proteome of Langat-Infected *Ixodes scapularis* ISE6 Cells: Metabolic Pathways Associated with Flavivirus Infection. PLoS Negl Trop Dis 2016, 10(2):e0004180.

2. Diamond DL, Syder AJ, Jacobs JM, Sorensen CM, Walters KA, Proll SC, et al. Temporal proteome and lipidome profiles reveal hepatitis C virus-associated reprogramming of hepatocellular metabolism and bioenergetics. PLoS Pathog 2010, 6(1):e1000719.

3. Pastorino B, Boucomont-Chapeaublanc E, Peyrefitte CN, Belghazi M, Fusai T, Rogier C, et al. Identification of cellular proteome modifications in response to West Nile virus infection. Mol Cell Proteomics 2009, 8(7):1623-1637.

4. Tchankouo-Nguetcheu S, Khun H, Pincet L, Roux P, Bahut M, Huerre M, et al. Differential protein modulation in midguts of *Aedes aegypti* infected with chikungunya and dengue 2 viruses. PLoS One 2010, 5(10).

5. Khadka S, Vangeloff AD, Zhang C, Siddavatam P, Heaton NS, Wang L, et al. A physical interaction network of dengue virus and human proteins. *Mol Cell* Proteomics 2011, 10(12):M111 012187.

6. Mitzel DN, Wolfinbarger JB, Long RD, Masnick M, Best SM, Bloom ME. Tick-borne flavivirus infection in *Ixodes scapularis* larvae: development of a novel method for synchronous viral infection of ticks. Virology 2007, 365(2):410-418.
